# Editorial: Human rights and inequity in health access of Central American Migrants

**DOI:** 10.3389/fpubh.2023.1104703

**Published:** 2023-02-07

**Authors:** Héctor Luis Díaz, Maria Elena Ramos-Tovar, Francisco Gonzalez-Salazar, Luis R. Torres-Hostos

**Affiliations:** ^1^School of Social Work, New Mexico State University, Las Cruces, NM, United States; ^2^School of Social Work and Human Development, Autonomous University of Nuevo León, San Nicolas de los Garza, Mexico; ^3^Mexican Social Security Institute (IMSS), Mexico City, Mexico; ^4^School of Social Work, The University of Texas Rio Grande Valley, Edinburg, TX, United States

**Keywords:** migrants, inequality, healthcare, photovoice, culturalism, access

*Frontiers in Public Health* is very pleased to publish this journal issue focusing on the health access of immigrants. Contributions to this journal issue include five articles that rely on different methodologies while focusing on diverse geographic world regions and target populations. This editorial summarizes these features while also highlighting the unique contributions of each article.

Stoesslé's article, “We Speak the Same Language but They do not Understand Us…” is the result of a qualitative research study based on 21 interviews with health and administrative staff in the Mexican State of Nuevo León. The study focuses on the use and abuse of “culturalism” in healthcare in this particular region. The main contribution of this article is the description of a paradox through which the well-intended efforts and desire of Mexican healthcare workers to provide “culturally competent” services often results in the creation of stereotypes of Central American migrants in transit through Mexico. These stereotypes, which often result from inadequate training in the area of culturally sensitive practice, commonly lead to erasing or ignoring the personal characteristics of migrants, inconsistent interpretations of the causes of their conditions, inappropriate treatments, and ultimately to subpar treatment outcomes.

Chavez-Baray et al.'s article, “*The Use of Photovoice Methodology to Assess Health Needs and Identify Opportunities Among Migrant Transgender Women in the U.S.-Mexico Border*,” is the result of a Participatory Action Research study that relies on “photovoice,” a specific qualitative research method. Reportedly, “photovoice” became an effective way to empower the 16 study participants who are transgender women migrating through the United States/Mexico border. Photographs and discussions with participants made it possible to identify four major themes and provide valuable insights into their experiences. Major themes include mental health, migration experiences and challenges, stigma, discrimination and resiliency, and impact of the COVID-19 pandemic. The article concludes with a “Call to Action” Plan and with a series of recommendations for public policy and evaluation. Migrant Transgender Women are among the most vulnerable of all migrant populations, and this article makes an important contribution to the literature.

Stallones et al.'s article, “*Colorado Agricultural Workers' Rights of Access to Key Healthcare Providers: A Policy Brief*,” is based on an analysis of Bill Section 8-13.5-202 in the North American State of Colorado. The article defines health as a human right and focuses on the need of agricultural workers to have access to medical, dental and behavioral healthcare at times and locations accessible to them. Key issues addressed by the bill include access to healthcare providers, access to transportation, and the need for more effective communication to agricultural workers of their rights. The article concludes by providing five actionable recommendations for improving the health and well-being of agricultural workers, many of whom are migrants.

In their article, “*Systematic Review of Integration and Radicalization Prevention Programs for Migrants in the U.S., Canada and Europe*,” Del Pino-Brunet et al. reviewed available and pertinent literature published before January 2019 involving programs aimed at promoting the social integration of migrants, with the expectation that such integration would prevent their engagement in radical behaviors. The authors reviewed 601 studies from which they selected 18 for inclusion in their study. They conducted full text analyses of these 18 studies. This enabled them to identify programs targeting migrant women, school-based programs, programs focusing on language acquisition, and programs aimed at providing migrants with support systems. Researchers identified four urgent areas of interest related to the psychosocial well being of migrants. These include acculturation stress and migratory morbidity, tension related to family dynamics, challenges related to the development of identities, and educational adaptations and results. The article identifies barriers to migrant social integration into their new host societies, and concludes with a series of recommendations for social policy.

Finally, in their publication “*The Writing's on the Wall: On Health Inequalities, Migrants, and Coronavirus*,” Shaaban et al. provide us with a conceptual article that describes the current inequalities experienced by migrant and ethnic groups in the United States, the European Union and England. Such inequalities extend to the areas of hospitalizations, mortality rates, access to services, and poorer health outcomes. They describe the experiences of African-Americans and Hispanics in the United States, as well as the experiences of migrants in the Old Continent. The authors identify barriers immigrants face in their efforts to access the healthcare systems of their respective host countries. These barriers include financial and administrative barriers, language limitations, cultural differences, and lack of knowledge regarding how the various healthcare systems operate. They discuss the issues of pre-existing health, social and socio-economic inequalities that include housing income, poverty, employment, and access to social security. The authors conclude their article by providing a comprehensive list of policy recommendations that have the potential to improve the lives and health outcomes of minority, immigrant, and vulnerable communities.

The articles included in this journal special issue clearly illustrate the value of research-informed practice and policy making, particularly when such research is conducted by a group of international scholars that complement each other in terms of their academic disciplines, research foci, countries of origin, and populations targeted by their research.

Using different methodological and theoretical approaches, the authors in this issue make important contributions to ongoing discussions about central concepts such as human rights, access to health, and social integration of people in mobility processes. While critical, access is not sufficient when daily services are rife with stigma, discrimination or stereotyping by health workers themselves and by the broader communities that see migrants leave (i.e., departure communities), travel through, or arrive (i.e., final destination communities). Moreover, when in the processes of integration into the final destination communities there are ongoing barriers to the development of new identities necessary for full social integration, health and social outcomes worsen. It is clear that it is necessary to insist on changes at the micro and macro level to achieve the true enjoyment of human rights by migrant individuals anywhere in the world.

Based on this diversity of publications, the need arises to implement tools that help offer a fairer and more equal treatment to all people who migrate in any part of the world, facing the inequalities that arise from discrimination based on race, gender, economic and social status. All human beings, migrants or not, should have these guarantees of equal and equitable treatment.

## Author contributions

This journal issue editorial was written primarily by HD, Director of the School of Social Work at New Mexico State University. All authors contributed to the article and approved the submitted version.

